# Long-Term Motor Learning in the “Wild” With High Volume Video Game Data

**DOI:** 10.3389/fnhum.2021.777779

**Published:** 2021-12-20

**Authors:** Jennifer B. Listman, Jonathan S. Tsay, Hyosub E. Kim, Wayne E. Mackey, David J. Heeger

**Affiliations:** ^1^Statespace Labs, Inc., New York, NY, United States; ^2^Department of Psychology, University of California, Berkeley, Berkeley, CA, United States; ^3^Helen Wills Neuroscience Institute, University of California, Berkeley, Berkeley, CA, United States; ^4^Department of Physical Therapy, University of Delaware, Newark, DE, United States; ^5^Department of Psychological and Brain Sciences, University of Delaware, Newark, DE, United States

**Keywords:** motor learning, video games, sensorimotor performance, motor acuity, eSports

## Abstract

Motor learning occurs over long periods of practice during which motor acuity, the ability to execute actions more accurately, precisely, and in less time, improves. Laboratory-based studies of motor learning are typically limited to a small number of participants and a time frame of minutes to several hours per participant. There is a need to assess the generalizability of theories and findings from lab-based motor learning studies on larger samples and time scales. In addition, laboratory-based studies of motor learning use relatively simple motor tasks which participants are unlikely to be intrinsically motivated to learn, limiting the interpretation of their findings in more ecologically valid settings (“in the wild”). We studied the acquisition and longitudinal refinement of a complex sensorimotor skill embodied in a first-person shooter video game scenario, with a large sample size (*N* = 7174, 682,564 repeats of the 60 s game) over a period of months. Participants voluntarily practiced the gaming scenario for up to several hours per day up to 100 days. We found improvement in performance accuracy (quantified as hit rate) was modest over time but motor acuity (quantified as hits per second) improved considerably, with 40–60% retention from 1 day to the next. We observed steady improvements in motor acuity across multiple days of video game practice, unlike most motor learning tasks studied in the lab that hit a performance ceiling rather quickly. Learning rate was a non-linear function of baseline performance level, amount of daily practice, and to a lesser extent, number of days between practice sessions. In addition, we found that the benefit of additional practice on any given day was non-monotonic; the greatest improvements in motor acuity were evident with about an hour of practice and 90% of the learning benefit was achieved by practicing 30 min per day. Taken together, these results provide a proof-of-concept in studying motor skill acquisition outside the confines of the traditional laboratory, in the presence of unmeasured confounds, and provide new insights into how a complex motor skill is acquired in an ecologically valid setting and refined across much longer time scales than typically explored.

## Introduction

Motor learning, the process of acquiring and refining skilled movements, is an essential human capacity (Fitts and Posner, 1979; Krakauer et al., 2019; Kim et al., 2020). For instance, motor learning enables babies to acquire walking and talking skills, and athletes to become champions. These motor skills are honed over a lifetime of practice (Crossman, 1959; Du et al., 2021), often demanding tremendous grit and intrinsic motivation (Ryan and Deci, 2000; Duckworth et al., 2007).

Fitts and Posner (1979) proposed a highly influential conceptual model of how motor learning progresses through three distinct stages (also see: Welford, 1968; Whiting et al., 1992): cognitive, associative, and autonomous. The cognitive stage entails learning what movements to perform. This stage is typically short yet effortful (Adams, 1982; Dickinson and Weiskrantz, 1985), and typically produces the steepest improvements in performance, as task-specific strategies are learned (McDougle et al., 2016). The association stage is characterized by learning to fine-tune how these movements should be performed. This stage is less effortful but occurs over a long period. Learners eventually reach the autonomous stage, when movements are performed not only accurately and rapidly, but also automatically and effortlessly.

The association stage yields the greatest improvements in motor acuity – the ability to execute actions more accurately, more precisely, and within a shorter amount of time (Müller and Sternad, 2004; Shmuelof et al., 2012, 2014; McDougle and Taylor, 2019; Wilterson, 2021). For instance, a baseball player who has learned all the fine-grained mechanical details of a baseball swing still requires continuous practice to consistently generate enough power to hit home runs. Movement kinematics, like the trajectory of the swing and the pivoting of the trunk, also improve in the association stage, becoming less variable and more coordinated over time (Logan, 1988; Deutsch and Newell, 2004; Liu et al., 2006; Hung et al., 2008; Müller and Sternad, 2009; Guo and Raymond, 2010).

Despite evidence for its importance, there is a paucity of studies examining improvements in motor acuity during the association stage of motor learning, a gap likely linked to the tight resource constraints on laboratory-based studies. Specifically, since in-lab studies are typically on the order of minutes to hours, studies on improvements in motor acuity that occur on the order of days to weeks (and even years) are rare (but see: Stratton, 1896; Semrau et al., 2012; Stafford and Dewar, 2014; Hardwick et al., 2019; Huberdeau et al., 2019; Berlot et al., 2020; Wilterson and Taylor, 2021). Moreover, the handful of studies that examine motor acuity have used relatively simple motor tasks (Flatters et al., 2014), like drawing circles as fast as possible within a pre-defined boundary (Shmuelof et al., 2012), throwing darts (Martin et al., 1996), or center-out reaching and grasping (Jordan and Rumelhart, 1992; Shadmehr and Mussa-Ivaldi, 1994). Whether insights generated in the lab can generalize to more naturalistic, ecological motor skills remains to be seen.

How then can we study the refinement of ecological, complex motor skills over longer timescales? There have been recent efforts to study motor skill acquisition outside the lab environment (Stafford and Dewar, 2014; Bönstrup et al., 2020; Drazan et al., 2021; Haar et al., 2021; Johnson et al., 2021; Tsay et al., 2021b). Online video game platforms, in particular, provide an unprecedented opportunity to reach more than 400 million intrinsically motivated people who voluntarily hone their cognitive, perceptual (Li et al., 2009; Appelbaum et al., 2013), and motor skills over weeks, months and even years (Green and Bavelier, 2003; Eichenbaum et al., 2014). Specifically, first person shooter games, where players use tools and weapons as intuitive extensions of themselves in a virtual 3D environment, provide an ideal testbed for studying motor skill acquisition over a long duration of practice (Stafford and Vaci, 2021). Successful shooters are those who can efficiently identify and localize relevant visual targets (Green and Bavelier, 2006), as well as rapidly and accurately hit their targets – a refined motor skill. Mining the wealth of motor performance metrics from these first-person shooter games, we may be able to better understand how complex motor skills are developed and refined over longer timescales.

We conducted an exploratory analysis of change in motor acuity and characterization of the factors influencing magnitude of learning over 100 days of gameplay using a commercial product, Aim Lab, that trains and assesses video game players to optimize their visuomotor performance in first-person shooter gaming scenarios. We have amassed a sizable dataset (>20 million players) with a wide range of abilities from beginner to professional esports athlete, from which we sampled pre-existing longitudinal data for this observational study (*N* = 7174 participants with over 62,170 game days of data). This study provides a proof-of-concept in studying motor skill acquisition in non-controlled conditions, outside the confines of the traditional laboratory using video game data.

## Materials and Methods

### Apparatus

Participants used the computer or laptop setup of their choice, likely in their home. Thus, we do not have information on make and model of computer, display, or mouse, display size, viewing distance, or chair height. We assume that there is a wide range of equipment combinations among participants.

### Task

Aim Lab is a commercial software product written in the C# programming language using the Unity game engine. Unity is a cross-platform video game engine used for developing digital games for computers, mobile devices, and gaming consoles (Brookes et al., 2020). Players download Aim Lab to their desktop or laptop PC. Players control their virtual weapon in Aim Lab tasks, using a mouse and keyboard, while viewing the game on a computer screen. Performance data are uploaded to Aim Lab secure servers.

Aim Lab includes over ninety different scenarios for skill assessment and training. Each exercise is tailored to a facet of first-person shooter gameplay, and can be customized to prioritize accuracy, speed, and other basic components of performance. Various scenarios assess and train visual detection, motor control, tracking moving targets, auditory spatial-localization, change detection, working memory capacity, cognitive control, divided attention, and decision making. Aim Lab players can perform a variety of tasks or play the same task repeatedly. It is common for players to log in more than once on a given day to play. Players do not pay for use of the game.

**FIGURE 1:**
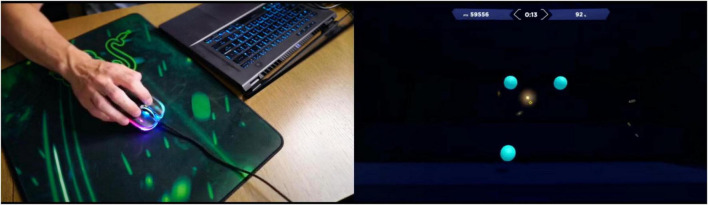
Gridshot task in Aim Lab. The 60-s run of Gridshot with three targets visible at all times is presented to a player on their computer screen **(Right)** while the player controls on-screen movements and shots with a standard computer mouse **(Left)**.

Of all task options, the “Gridshot” task has the highest median number of repeats per player within the same day. This 60-s task assesses motor control of ballistic movements. Three targets are presented simultaneously, at any given time, with a new target appearing after each target is destroyed. All targets are the same size, ranging between 1.3° and 1.7° (deg of visual angle), assuming a range of viewing distances and a range of values for the field of view in the virtual environment of the game (set by the player). New target appearance locations are randomized to one of 25 positions in a 5 × 5 grid, ranging between 4.8° and 9.1° wide and 5.1° and 7.8° high, again depending on viewing distance and field of view. The player destroys a target by moving their mouse to aim and clicking the left mouse button to shoot (Figure 1 and demonstration video of Gridshot^1^). Because multiple targets are present at once, combined with unlimited target duration and no explicit incentive to destroy any specific target, the player themself must decide the order in which to destroy the targets.

Players receive immediate feedback upon target destruction; an explosion sound is emitted, and the orb-shaped target splinters into multiple pieces and then disappears. Players receive summary feedback after each 60-s run of Gridshot, including score, hits per second (number of targets successfully destroyed per second), and hit rate (% of shot attempts that successfully hit a target). Points are added to the score when targets are hit and subtracted for shots that miss a target, and score is displayed at the top of the screen throughout the run. These metrics are automatically calculated in the game’s software, written in Unity, and displayed to the player as well as sent to a secure server. The number of points added for each target hit are scaled by time since the previous target hit. That is, more points are added when the time from the previous hit is shorter. Thus, players are incentivized to shoot targets rapidly and accurately as well as quickly plan their next movement and shot. While players are shown multiple metrics at the end of each run, it is likely that they are consciously optimizing for increased score. Some players compete for high scores on a leaderboard, which displays top scores across all players.

Data were generated through self-directed, typical use of the software on the players’ own gaming equipment in the setting and times of their choosing. Participants were not provided with instructions other than those available in the game. For a player of first-person shooter games, the tasks in Aim Lab are typically intuitive, since they mimic first-person shooter gaming scenarios.

### Participants

Historical (pre-existing) longitudinal data were sampled from a subset of 100,000 randomly selected Aim Lab player IDs from those who signed up for an account on or after 7/1/20 and who played on more than one date between then and 1/30/21. Participants were not given instructions to play any specific Aim Lab tasks nor to play for a specific length of time. Aim Lab is a free to play game and participants were not compensated for use of their play data, as allowed through Aim Lab’s terms of service. Play data were initially acquired for commercial purposes, are stored separately from player account data and without personal identifiers; thus, informed consent is not required for this study (Advarra Institutional Review Board).

Aim Lab players are located throughout North, Central, and South America, Africa, Europe, Asia, Australia, and The Middle East, based on gaming device IP addresses’ approximated locations. Players can view Aim Lab instructions and results screens in the language of their choice; the game has translations in over twenty languages. Since this study relied solely on pre-existing data from Aim Lab use and players were not contacted nor asked to fill out gaming equipment, gaming habit, demographic, or health-related questionnaires, we have no information on participants’ equipment, other video games they play or how long they play them, gender, age, health status, prescription or non-prescription drug use, medical conditions, disorders, or diagnoses, exposure to toxic substances, mobility constraints, injuries, use of assistive technologies, motivation for using Aim Lab, level of competitiveness, personality traits, sleep, caffeine intake, or exercise habits. Thus, participants were not excluded or included according to any criteria other than the volume of data they produced. Aim Lab does not collect personal or demographic information from players when they create an account. Based on an unrelated in-game survey completed by 4700 Aim Lab players (not necessarily overlapping with those whose data were analyzed here), we estimate that the player base is approximately 90% male, with a median age of 18 years and range of 13–70 years. We assume that the participant pool included in this study has similar gender and age characteristics and a world-wide geographic distribution.

### Data Analysis

We downloaded all Gridshot data for runs logged by the randomly selected players between 7/1/2020 and 1/30/21. Data for each 60-s run of Gridshot included player ID (a unique code for each player), date and time, mode (practice or compete), score, hits per second, and hit rate.

We limited our analysis to runs played with the weapon type “pistol,” the most popular weapon used, and the analysis was limited to players who played more than 1 day. Game days greater than 100 and with fewer than three Gridshot runs were removed. We removed Gridshot runs with missing values, possible equipment problems (ex: date/time stamps in the future), or indicative of cheating (proprietary Aim Lab methods for identifying cheaters).

**FIGURE 2:**
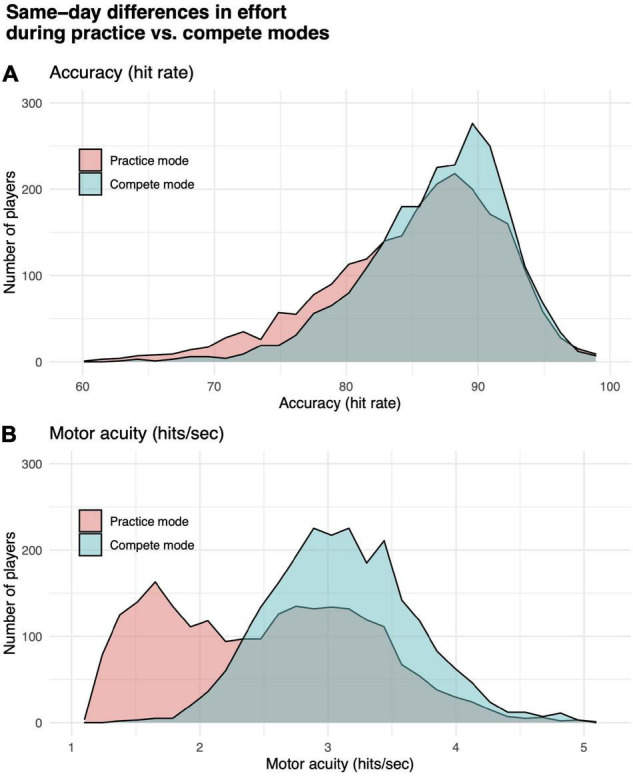
Differences in effort while playing in practice vs. compete modes. **(A)** Motor performance accuracy (hit rate). **(B)** Motor acuity (hits per second). Distributions of performance in practice (orange) vs. compete (teal) mode for a subset of participants who played Gridshot in both modes on the same day.

We tested for a significant difference between performance in practice vs. compete modes. Gridshot can be played in either mode. Results from runs played in compete mode are counted toward a player’s position on a leaderboard, which is visible to other players, while results from runs in practice mode are visible only to the player. We hypothesized that players would put out more effort and pay more attention to the task if they knew their scores were going to be visible to other players (compete mode) compared to their behavior in practice mode. It is known that reliability of performance assessments can be confounded by “effort” or level of attention the subject is paying to the assessment (Green et al., 2001). Paired sample *t*-tests showed that players (*N* = 2303, not necessarily overlapping with subsequent analyses reported below) using both practice and compete modes in Gridshot on the same day performed statistically significantly worse while in practice mode compared to compete mode (hits per second: *t* = 38.83, *df* = 3202, *p*-value < 1e-6; hit rate: *t* = 14.44, *df* = 3202, *p*-value < 1e-6). Differences in distributions of hits per second and hit rate by mode are shown in Figure 2. While Gridshot runs performed in practice mode may not be performed with maximal effort, we assume that this practice time could still contribute to learning. Therefore, for all further analyses, a given player’s total runs of Gridshot per day (*K*) or across days between 7/1/20 and 1/30/21 (*L*) were based on Gridshot runs in either practice or compete mode, while performance metrics (hit rate and hits per second) were limited to runs played in compete mode, only.

Although we restricted our analyses to Gridshot runs played in compete mode, excluding those played in practice mode, after demonstrating the statistically significant difference in “effort” between modes, it is still possible for participants to play in compete mode with low effort. For example, players might be interrupted during play and fail to complete a 60 s run in compete mode. We calculated the median score for all players on each day in compete mode, then calculated the ratio of this median day score to the median score on that player’s first day of game play, and then calculated the mean and standard deviation of this ratio. We then removed data (all of a player’s runs on any given day) where this ratio was less than 2 SD below its mean, which was equivalent to median day score <80% of the player’s median score on their first day playing Gridshot, because we assume this indicates low effort.

Our inclusion criteria resulted in 62,170 days of play from 7174 players totaling 682,564 Gridshot runs (60 s each). Because score is a function of both hits per second and hit rate, we limited our analyses to hits per second and hit rate.

For each player we calculated:

• L runs total (between 7/1/20 and 1/30/21)
• *M* days played (between 7/1/20 and 1/30/21)
• *K* runs per day
• Median of *K* runs per day, based on all days played

For each player’s first day playing Gridshot we calculated:

• Start date (calendar date of first run of Gridshot)
• Baseline score (from run 1 on game day 1)
• Baseline hit rate (from run 1 on game day 1)
• Baseline hits per second (from run 1 on game day 1)

On each successive game day we calculated:

• Days from last play (*M* days between calendar dates of play)
• Day number (the *m*^th^ day the player played the game)
• Score, hit rate, and hits per second for each run

**FIGURE 3:**
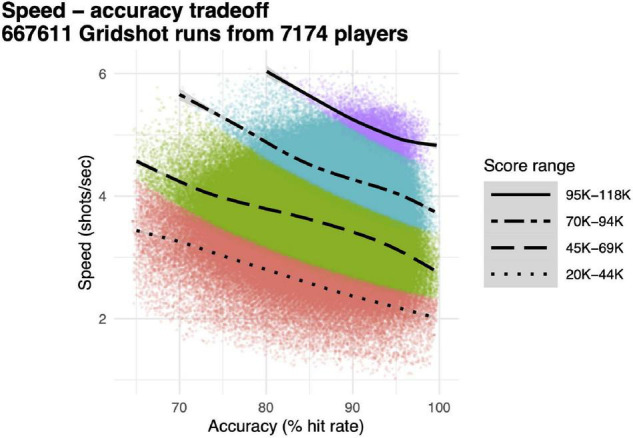
Speed–accuracy tradeoff. Individual data points, speed (shots per second, *y*-axis) and accuracy (hit rate, *x*-axis) for each individual 60 s run of Gridshot. Colors and line-types, score quartiles. Curves, locally weighted regression fits per score quartile.

Our main dependent variable for performance accuracy was hit rate, proportion of shots that hit targets, and our main dependent variable for motor acuity was hits per second, the product of speed (attempted shots per second) and performance accuracy (hit rate). Speed and accuracy were computed separately for each 60 s run of Gridshot (Figure 3, individual data points), along with score (Figure 3, colors). Each quartile of score exhibits a speed–accuracy tradeoff; speed is higher when accuracy is lower and vice versa. Runs with higher scores have correspondingly both higher speed and higher accuracy. Motor acuity increases as the product of speed and accuracy. This is represented in Figure 3 along the 45° diagonal, up and to the right.

Our dependent variables were assessed for test–retest reliability. We calculated intraclass correlation, ICC(A,1) absolute agreement between performance on consecutive days of play, from play days 1:2 to play days 28:29 for 200 randomly selected players, based on three and separately on five randomly selected runs per day per player, using the irr package in R (parameters model = “twoway,” type = “agreement,” unit = “single”) (Gamer et al., 2019). Consecutive days of play are not necessarily consecutive calendar days, since players often skip days between play.

#### Does Motor Performance Accuracy and Motor Acuity Improve Across Days of Gameplay?

Specifically, we asked how hit rate, our measure of performance accuracy, and hits per second, our measure of motor acuity, improved across the number of days of gameplay. Since the total number of days played differs by player, the sample sizes for each data point were uneven. For example, on Days 1 and 2, *N* = 7174 on Day 60, *N* = 82.

#### How Does the Amount of Practice Affect Motor Performance Accuracy, Motor Acuity, and Retention?

Specifically, we asked how the amount of learning on day *m*, defined as the number of runs on a given game day, impacted the player’s improvement in performance from baseline (i.e., hits per second on run 1 of day 1).

The number of players decreased with each subsequent run and each subsequent day, because some players stopped playing. Consequently, to examine patterns of learning within a day and retention of learning to the following day, the data were filtered to exclude performance data from days for which the previous calendar date had been skipped and where *N* players <200 for each data point. The remaining data set included 5144 players’ Gridshot data. For ease of exposition, we limited our analysis of learning within a day to runs 1–25 on days 1–5. Since different players played different numbers of runs on each day, the sample sizes for each data point were uneven (Day 1, *N* = 5144; Day 5, *N* = 221).

We also asked how the amount of learning on day *m* − 1 impacted retention on day *m*. Retention was calculated as 100(*S*_*m*,1_−*S*_*m*−1,1_)/(*S*_*m*−1,25_−*S*_*m*−1,1_), where *S*_*m,k*_ is the increase in score from baseline for run *k* on day *m*. Thus, retention, as operationally defined here, represents the magnitude of learning (performance improvement) that is retained from the end of 1 day to the start of the next, normalized by the previous day’s total magnitude of learning. We restricted the analysis to consecutive days of game play within days 2 through 21, and furthermore to consecutive days with *N* ≥ 20 players. We removed outlier values (prob > 0.95) of the resulting retention metric, for a given day *m*, based on median absolute deviation using the outliers package in R (Komsta, 2011). We removed outliers because retention was calculated as a ratio which exhibited statistical anomalies. We then fit the relationship between retention and day number with a non-parametric regression model that fits a polynomial function, based on local fitting (locally weighted scatter plot smoother a.k.a. loess regression) (Cleveland, 1979; Komsta, 2011), using the loess function from the stats package in R with the R function default values for the degree (the degree of the polynomials to be used in the regression) and span (size of the neighborhood of points in which smoothing takes place) parameters (degree = 2, span = 0.75) and plotted the results (R Core Team, 2021).

#### How Much Should Gamers Practice on a Given Day to Maximize Improvements in Motor Acuity?

Specifically, we characterized how improvements in hits per second from run *k*, day *m* to run *k*, day *m* + 1 for *k* = 1, 2, and 3, varied as a function of the number of runs on day *m*. After calculating improvement per player from 1 day to the next, we removed outlier values (prob > 0.95) of improvement, for a given value of *k*, based on median absolute deviation (Komsta, 2011). For each value of *k* (1–3), we fit the relationship between number of runs the previous day and median change in hits per second using the loess function from the stats package in R (degree = 2, span = 0.75) and plotted the predicted values based on the model. We restricted our analyses to runs up to 100 on days 1–21 (first 3 weeks) of game play to maintain a large player sample size per data point. The remaining data set included Gridshot data from 3461 players.

#### Exploring Which Factors of Gameplay Contribute to Improvements in Motor Acuity

To characterize the phenomena observed with the methods described above and to understand the possible contributions of various factors to motor learning over time, within the context of this first-person shooter task, we fit repeated measures linear mixed models (growth model), separately for hits per second and hit rate as outcome variables. The models accounted for repeated (correlated) measures within players as well as different performance levels and practice behaviors between players.

Data were limited to Gridshot runs with day number ≤60 to maintain a large sample size for each day number in the test set. Based on pilot analyses indicating that improvements are non-linear, we transformed day number and median runs per day into log(day number) and log(median runs per day). Fixed effects included start values for each performance metric on day 1, log(median runs per day), days from last play, log(day number), and interactions between all fixed effects. We allowed for random intercepts and slopes for each player. Due to the large sample size of the training data set (*N* players = 5712), tests for normality of outcome measures were not necessary.

We built and validated a pair of models (one for hit rate and one for hits per second) *via* backward stepwise elimination followed by cross-validation. Data were split into train and test sets (80/20) using the groupdata2 package in R (Olsen, 2021), evenly distributing players based on total days played. We started with model variants that included maximal combinations of regressors. These models were fit to the training data (*N* players = 5712) using the R package lme4 (algorithm “REML,” optimiser “nloptwrap,” link function “identity,” family “gaussian”) (Bates et al., 2015; Kuznetsova et al., 2017). The R package lmerTest was used to perform backward stepwise elimination from each maximal model to reach a final model based on likelihood ratio tests. The final pair of models were validated using the test set (*N* players = 1427). Sample R code (Supplementary File 1) and dummy train and test sets (Supplementary Files 2, 3) can be found in Supplementary Material.

To evaluate predictor variables for multicollinearity, prior to model testing, correlation coefficients among all potential predictor variables were calculated. All pairwise correlation coefficients were −0.07 to +0.07. Generalized mixed-effects models employ both marginal and conditional *R*^2^ metrics, where marginal *R*^2^ refers to the amount of variance explained only by fixed effects (here, fixed effects refers to day number, start value, days skipped, and number of repeats) and conditional *R*^2^ refers to the amount of variance explained after adding in random effects (here, random effects refers to varying intercept and slope among players). Marginal and conditional *R*^2^ were similar for train and test sets for each of the two models developed to explain factors that contribute to variance in motor learning within the context of Gridshot, indicating that the models were not over-fitted (Table 1). The resulting model structures are reported in Table 2, which contains main and interaction effect coefficient estimates (“Estimates”) as a measure of their individual effect size on the outcome measures, confidence intervals, and a *p*-value *via* Satterthwaite’s degrees of freedom method. Table 2 includes marginal and conditional *R*^2^ for mixed models (Nakagawa and Schielzeth, 2013), and variance (among players) computed in the R package sjPlot (Lüdecke, 2021).

**TABLE 1:** Model validation.

Model	Conditional *R*^2^	Marginal *R*^2^	RMSE
HPS train	0.93	0.71	0.13
HPS test	0.94	0.72	0.13
Hit rate train	0.80	0.26	0.22
Hit rate test	0.81	0.25	0.22

**TABLE 2:** Model performance.

	Median day hps			Median day hit rate		
Predictors	Estimates	CI	p	Estimates	CI	p
(Intercept)	0.74	0.71 – 0.78	**<0.001**	1.35	1.29 – 1.40	**<0.001**
start_hps	0.79	0.77 – 0.81	**<0.001**			
log(day_number)	0.24	0.23 – 0.26	**<0.001**	0.34	0.31 – 0.36	**<0.001**
log(median_runs)	0.1	0.07 – 0.14	**<0.001**	0.04	0.02 – 0.06	**<0.001**
days_fromlastplay	0	−0.01 – 0.00	0.13	−0.05	−0.06 – −0.04	**<0.001**
start_hps * log(day_number)	−0.06	−0.07 – −0.05	**<0.001**			
start_hps * log(median_runs)	−0.03	−0.05 – −0.00	**0.018**			
log(day_number) * log(median_runs)	0.04	0.03 – 0.06	**<0.001**	0.01	0.01 – 0.02	**<0.001**
start_hps * days_fromlastplay	0	−0.01 – −0.00	**0.003**			
log(day_number) * days_fromlastplay	−0.01	−0.02 – −0.01	**<0.001**	−0.03	−0.04 – −0.03	**<0.001**
log(median_runs) * days_fromlastplay	−0.01	−0.02 – −0.00	**0.001**	0	0.00 – 0.00	**0.043**
[start_hps * log(day_number)] * log(median_runs)	−0.01	−0.02 – −0.00	**0.005**			
[start_hps * log(day_number)] * days_fromlastplay	0	0.00 – 0.00	**0.044**			
[start_hps * log(median_runs)] * days_fromlastplay	0.01	0.00 – 0.01	**0.007**			
[log(day_number) * log(median_runs)] * days_fromlastplay	−0.01	−0.01 – −0.00	**0.001**			
[start_hps*log(day_number)*log(median_runs)] * days_fromlastplay	0	0.00 – 0.01	**0.007**			
start_hit_rate				0.39	0.36 – 0.42	**<0.001**
start_hit_rate * log(day_number)				−0.15	−0.16 – −0.14	**<0.001**
start_hit_rate * days_fromlastplay				0.01	0.01 – 0.02	**<0.001**
[start_hit_ratelog * (day_number)] * days_fromlastplay				0.01	0.01 – 0.01	**<0.001**
Random effects						
σ^2^	0.02			0.06		
τ_00_	0.06 player ID			0.15 player ID		
τ_11_	0.01 player ID.log(day_number)			0.02 player ID.log(day_number)		
ρ_01_	0.74 player ID			0.65 player ID		
ICC	0.77			0.73		
*N*	5712 player ID			5712 player ID		
Observations	42635			42635		
Marginal *R*^2^/conditional *R*^2^	0.71/0.93			0.26/0.80		

*Bold values denote statistical significance at the p < 0.05 level.*

## Results

### Test–Retest Reliability

**FIGURE 4:**
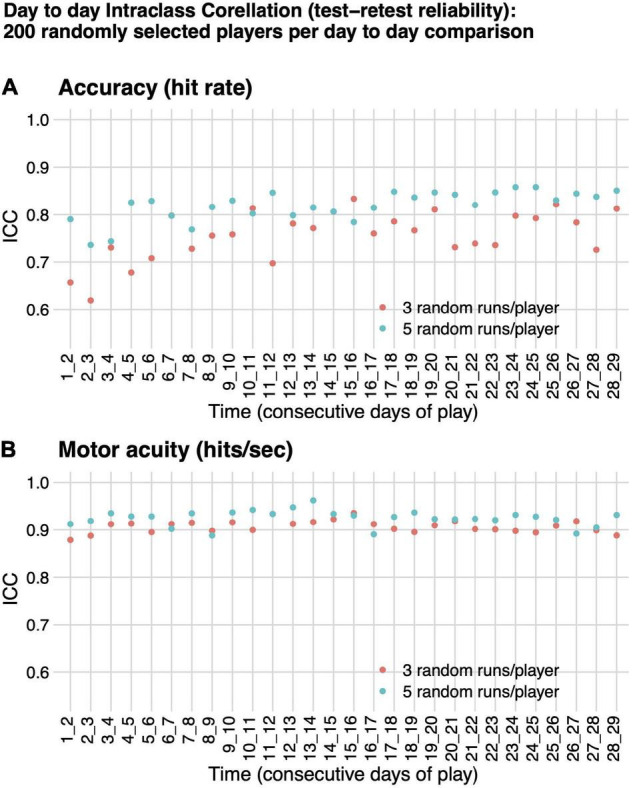
Intraclass correlation (test–retest reliability). ICC(A,1) absolute agreement between performance on consecutive days of play for 200 randomly selected players, based on either three (orange) or five (teal) randomly selected runs per day per player. **(A)** Motor performance accuracy (hit rate). **(B)** Motor acuity (hits per second).

For all pairs of consecutive days of play evaluated, test–retest reliability, as measured by ICC(A,1) was higher for motor acuity (hits per second) than for motor performance accuracy (hit rate), although both had ICC as high as or better than many cognitive or motor assessments in healthy adults (Figure 4; Broglio et al., 2018; Lima et al., 2018). ICC(A,1) for motor acuity ranged from 0.89 (*F* = 17.01, *df* = 199, *p*-value < 1e-6) to 0.96 (*F* = 54.27, *df* = 199, *p*-value < 1e-6) when based on five runs of Gridshot and from 0.88 (*F* = 16.87, *df* = 199, *p*-value < 1e-6) to 0.94 (*F* = 30.71, *df* = 199, *p*-value < 1e-6) when based on three runs of Gridshot. ICC(A,1) when based on motor performance accuracy ranged from 0.74 (*F* = 6.62, *df* = 199, *p*-value < 1e-6) to 0.86 (*F* = 13.08, *df* = 199, *p*-value < 1e-6) for five runs of Gridshot and from 0.62 (*F* = 4.32, *df* = 199, *p*-value < 1e-6) to 0.83 (*F* = 10.97, *df* = 199, *p*-value < 1e-6) when based on three runs of Gridshot.

### Does Motor Performance Accuracy and Motor Acuity Improve Across Days of Gameplay?

We used hit rate, percentage of shot attempts that successfully hit a target, to measure how motor performance accuracy improved over 60 days of gameplay. A greater hit rate indicated that the player was more accurate in hitting the target. Players’ hit rate ranged from 65.17% to 99.12% on day 1 (*N* = 7174) (mean = 85.70, SD = 5.52) and from 65.43% to 99.22% across all other days (*N* = 7174 on day 2 to *N* = 82 on day 60) (mean = 86.15, SD = 4.91), exhibiting a wide distribution among players. This heterogeneity in performance was likely attributed to the wide range of gaming experience among players on the Aim Lab platform, which ranged from amateur to professional. Summary statistics for plays in compete mode are shown in Table 3.

**TABLE 3:** Player summary statistics (*N* = 7174).

	Mean	SD	Median	Mad	Min	Max	Skew	Kurtosis	SE
Total runs across all days	142.61	257.94	56	57.82	6	3707	5.03	37.1	3.05
Total days played	14.66	17.79	8	7.41	2	150	2.85	10.26	0.21
Start (day 1) hit rate	85.7	5.52	86.17	5.56	65.17	99.12	−0.51	0.17	0.07
Start (day 1) hits per second	2.72	0.47	2.72	0.46	1.23	4.89	0.19	0.51	0.01
Days from last play	8.01	17.78	2	1.48	1	202	4.84	29.78	0.21
Runs per day	9.35	8.64	7	4.45	3	156	4.54	38.94	0.1
Hit rate	86.15	4.91	86.64	4.72	65.43	99.22	−0.6	0.54	0.06
Hits per second	2.95	0.5	2.93	0.49	1.27	5.07	0.15	0.27	0.01

**FIGURE 5:**
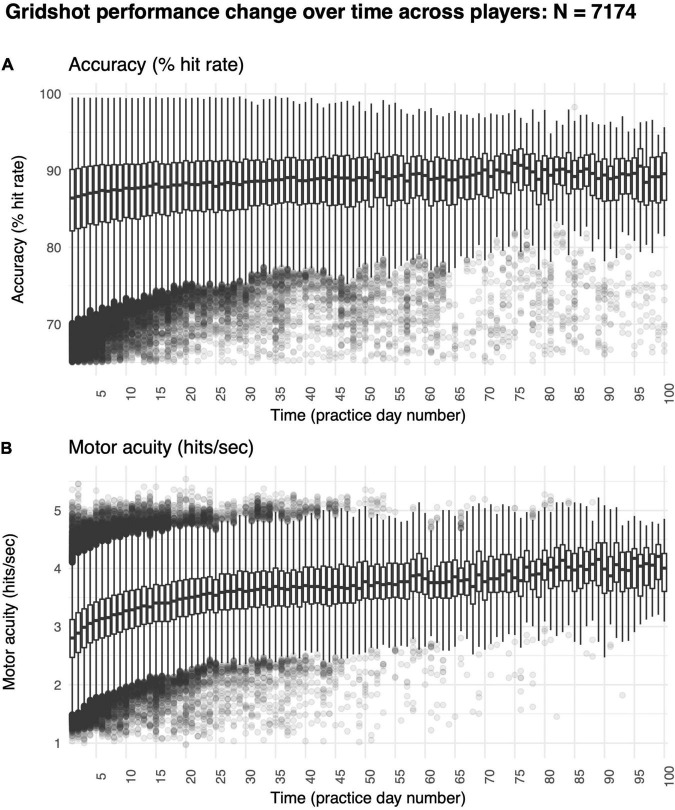
Improvements in motor skill over 60 days of shooting practice. **(A)** Changes in motor performance accuracy (hit rate: percentage of shots attempted that hit targets) and **(B)** motor acuity (hits per second: number of accurate shots that hit targets every second). Box plots denote the median (thick horizontal lines), quartiles (1st and 3rd, the edges of the boxes), extrema (min and max, vertical thin lines), and outliers (Q1 +/− 1.5*IQR, points).

We found a significant increase in hit rate over time (Figure 5A). Specifically, median hit rate on day 1 was 86.10% and on day 60 was 89.60% (*t* = −5.09, *df* = 83.95, *p*-value < 1e-6). Improvements in hit rates were also non-linear (*R*^2^ non-linear: 0.02, *R*^2^ linear: 0.02), perhaps because many players started to reach a ceiling level of performance. The average rate of increase was 0.03% per day, exhibiting a very modest rate of improvement in motor performance accuracy over time.

Improvements in hit rate, however, may not be the most sensitive measure of motor learning for two reasons. First, measures of performance accuracy may readily hit a ceiling or a floor – a concern that is also present in our own dataset. Second, because changes in performance only occur early in learning, they may measure improvements in task-irrelevant dimensions, such as familiarization with which button to click or even posture in the gaming chair, rather than improvements in the motor skill of interest (e.g., shooting a target).

Measures of motor acuity, like hits per second (number of targets successfully destroyed per second), offer a more sensitive measure of motor learning. These measures typically exhibit a greater range of improvement both early and late in learning, and as such, can better capture improvements in motor execution rather than only improvements in task-irrelevant dimensions. Median hits per second ranged from 1.23 and 4.89 on day 1 (mean = 2.72, SD = 0.47) and from 1.27 to 5.07 across all other days (mean = 2.95, SD = 0.50), also exhibiting large heterogeneity amongst players. To put this range in perspective, ten of the recent top Gridshot performers in Aim Lab had a median hits per second of 6.25, indicating that the median performance among players in our sample is far from ceiling.

Most critically, we found large improvements in motor acuity over time (Figure 5B). Specifically, the median hits per second was 2.72 on day 1, whereas it was 3.78 on day 60 (*t* = −21.27, *df* = 83.95, *p*-value < 1e-6). Improvements in hits per second were non-linear (*R*^2^ non-linear: 0.21, *R*^2^ linear: 0.26), showing a trend similar to in-lab studies studying motor skill acquisition (Telgen et al., 2014; Yang et al., 2021) and validating our online approach.

Together, we observed improvements in both motor performance accuracy and motor acuity during first-person video gameplay, providing a proof-of-concept in studying motor learning outside the lab.

### How Does the Amount of Practice Affect Motor Performance Accuracy, Motor Acuity, and Retention?

**FIGURE 6:**
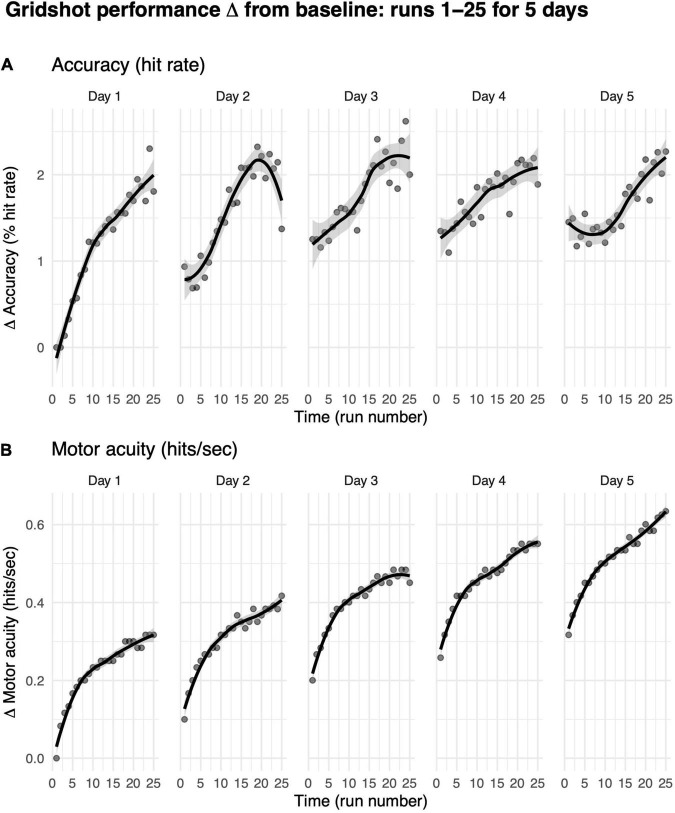
Motor performance exhibits within-day and across-day improvements. **(A)** Motor performance accuracy (hit rate). **(B)** Motor acuity (hits per second). Curves, locally weighted regression fitted to the data. Shaded error bars denote the 95% confidence interval. Performance up to 25 runs over a maximum of 5 days, limited to days on which the player also played the previous day.

We first asked how practice influences how much gamers improve in their motor performance accuracy (i.e., hit rate) from their baseline performance on their first run on day 1. We limited our analyses to players who completed five consecutive days of shooting practice with at least 25 runs on each day (each data point in Figure 6 represents more than 200 players).

Hit rates increased from baseline within each game day (Figure 6A). On day 1, for instance, within-day performance change from baseline per run increased significantly from 0% on run 1 (baseline, by definition) to 2.09% on run 25 (*t* = −8.71, *df* = 638, *p*-value < 1e-6). The rate of increase on day 1 was moderate, exhibiting a 0.09% improvement in hit rate per run. These data suggest that more practice runs within the same gameday increase shooting accuracy.

We then asked whether performance improved across these 5 days. Average improvements in hit rate increased across days 1 to 3 (day 1: 0.23 ± 2.12, day 2: 1.25 ± 6.16, day 3: 1.6 ± 6.58.), but there was no evidence for any additional increase from days 3 to 5 (*t* = −0.40, *df* = 3761.70, *p*-value = 0.35). That is, average hit rate increase on each day plateaued, exhibiting an asymptotic mean improvement in hit rate of 1.70% ± 6.37%. While these data suggest that gamers were becoming more accurate shooters with greater within and across-day practice, motor performance accuracy seemed to saturate, hitting an upper bound with as little as 5 days of practice. These data also highlight how hit rate cannot capture the richer aspects of motor learning that occur over a prolonged period of practice (e.g., 60 days, see Figure 5A).

Changes in motor acuity (i.e., measured by hits per second) provided a better, more sensitive index of motor skill acquisition both within each day of practice and across days of practice (Figure 6B). On day 1, change from baseline in hits per second increased significantly from 0 (baseline, by definition) to 0.32 (*t* = −27.13, *df* = 638, *p*-value < 1e-6). That is, players were able to hit one more target roughly every 3 s. These within-day improvements increased with more practice runs in a non-linear manner [marginal *R*^2^ linear (0.12) < marginal *R*^2^ non-linear (0.32)]. The rate of increase on day 1 was quite fast, exhibiting a 0.01 hits per second improvement per run. Indeed, 25 runs of practice helped gamers hit targets more accurately and more rapidly.

There was a rapid improvement in hits per second over the first few runs on each successive day (Figure 6B). We hypothesize that this “warm-up” effect is, in part, due to motor adaptation (see the section “Discussion”).

Across days, change from median hits per second on the first run to the 25th run on each day increased from 0.32 ± 0.30 on day 1 to 0.63 ± 0.35 on day 5. In contrast to hit rate, motor acuity improvement did not show signs of plateauing; instead, hits per second continued to improve over each day.

**FIGURE 7:**
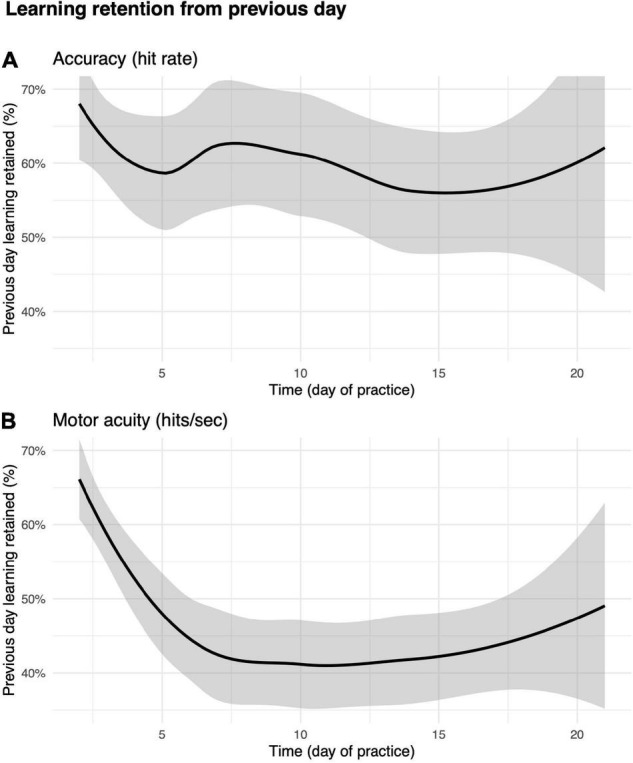
Percent improvement on day *m* retained on day *m* + 1. **(A)** Motor performance accuracy (hit rate). **(B)** Motor acuity (hits per second). Curves, locally weighted regression fitted to the data. Shaded error bars denote the 95% confidence interval.

We then asked how much of the improvements in motor performance accuracy and motor acuity were retained across days? As shown in Figures 7A,B, the first run on day *m* + 1 was greater than the first run on day *m*, suggesting that some of the performance/acuity gains were retained (see the section “Materials and Methods” for details).

Performance accuracy (hit rate) retention was greatest in the early days of practice (Figure 7A). For instance, participants were able to retain as much as 67% ± 79% improvements in hit rate from day 1 to day 2. The percent value of retention did not decrease drastically over subsequent days. We next examined how motor acuity (hits per second) was retained from 1 day to the next (Figure 7B). On day 2, players retained 65% ± 52% of what they learned from day 1. Percent retention declined across days, plateauing at 40% after day 10. This effect not only implies that motor acuity was still exhibiting within-day improvements (also supported by Figure 6B), but also that improvements in motor acuity could be carried over to the next day.

### How Much Should Gamers Practice on a Given Day to Maximize Improvements in Motor Acuity?

While more practice on a given day may yield greater improvements in hits per second, does more practice always mean that these improvements are retained? Here, we reasoned that there could be a sweet spot in motor learning. Whereas too little practice would be detrimental to retention since little was learned in the first place, too much practice may also be detrimental due to mental/physical fatigue (Branscheidt et al., 2019). To explore this question, we calculated how much motor acuity improved across days *m* to *m* + 1 as a measure of how long gamers practiced on day *m*.

**FIGURE 8:**
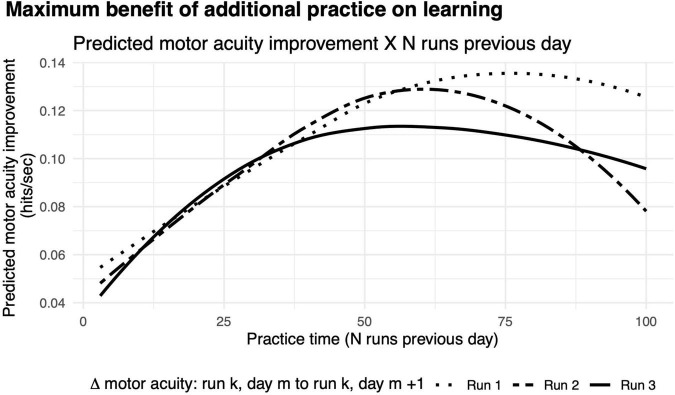
Diminishing returns on motor acuity with more practice on the previous day. Curves, model predictions for improvements in motor acuity (hits per second) on run 3 (solid), run 2 (dashed), and run 1 (dotted) as a function of number of runs in the previous day.

We found that the benefit of additional practice exhibited a non-monotonic function (Figure 8). The greatest improvements in motor acuity were evident with 75 runs when comparing the first run on two consecutive days, and 90% learning benefit was achieved by practicing 49 runs, which roughly corresponds to 50 min of gameplay. A similar pattern held when comparing performance from run 2 and run 3 on two consecutive days, showing maximum benefit of learning with 60 (90% benefit after 41 runs) and 57 runs of practice (90% benefit after 33 runs), respectively. Taken together, this non-monotonic function provides strong evidence for diminishing returns on practice, similar to those of previous studies (Stafford and Dewar, 2014). As noted above, we observed a rapid improvement in motor acuity over the first few runs on each successive day (Figure 6B), a “warm-up” effect that we hypothesize is due to motor adaptation (see the section “Discussion”).

After three runs of warm-up on every day, the maximal improvement in motor acuity across days is seen with about an hour of practice (Figure 8, dashed curve). With no warm-up, considerably more practice (75 min) on any given day is needed to achieve maximal benefit the following day (Figure 8, solid curve). We infer that the additional practice is needed to compensate for the lack of warm-up.

### Exploring Which Factors of Gameplay Contribute to Gamers’ Improvements in Motor Acuity

We took an exploratory approach to the data by building the most parsimonious models (*via* stepwise regression and cross-validation), using all known, measurable factors of gameplay to explain their contributions to gamers’ improvements in motor performance accuracy (hit rate) and motor acuity (hit rate per second) over time.

The best-fitting model that converged and best explained change in performance accuracy (hit rate) included main effects for baseline performance accuracy (i.e., median hit rate on day 1), number of days of practice [log(day number)], amount of practice within a day [log(median runs per day)], and number of days skipped between practice days, with interactions effects between baseline performance accuracy × log(day number), baseline performance accuracy × days from last play, log(day number) × days from last play, log(day number) × log(median runs per day), baseline performance accuracy × log(day number) × days from last play. Because each player started playing with a different level of performance accuracy, we allowed each player to have their own slope and intercept (Table 2).

The significant negative interaction effect of day number on baseline motor performance accuracy indicates the better a player’s starting hit rate, the less their hit rate increased per additional day of practice; or, the magnitude of the positive effect of a unit increase in start hit rate on predicted learning was decreased by each successive day number. While skipped days (days from last play) had statistically significant interaction effects with other variables, along with that of the main effect for skipped days, the effect sizes (coefficients) were quite small, meaning skipping days between practice sessions has a small negative effect on motor performance accuracy that could likely be overcome with additional practice.

To provide some intuition behind our model, we illustrate how our model predicts motor performance accuracy for a typical player. A typical player with modest accuracy (a baseline hit rate of 85.67% on day 1), who plays 10 min of Gridshot recreationally per day (10 runs, without skipping any days of practice), may be predicted to have a hit rate of 86.50% on day 10, 86.73% on day 20, 86.85% on day 30, and 86.94% on day 40. If instead they played 20 runs of Gridshot per day, the same player would have predicted values of 86.82% hit rate on day 10, 87.13% on day 20, 87.31% on day 30, and 87.43% on day 40. The predicted improvements in motor performance accuracy are quite modest.

The model that best explained change in motor acuity (hits per second) included main effects for baseline motor acuity (i.e., median hit rate on day 1), number of days of practice [log(day number)], amount of practice within a day [log(median runs per day)], number of days skipped between practice days, and all potential interaction effects, with independent slope and intercept for each player (Table 2).

As was the case for hit rate, there was a significant negative interaction effect of day number on start value for hits per second, meaning the greater a player’s starting value for hits per second, the less increase there was in motor acuity per additional day of practice. Similarly, there was a negative coefficient for the interaction effect between median runs per day and start value for hits per second, implying that additional practice within a day had less of an effect on the magnitude of learning for players who were more skilled to begin with. Again, skipped days (days from last play) had statistically significant interaction effects with other variables in the explanatory model, as well as a statistically significant main effect, but with negligible coefficients.

For example, a typical player with a start value of 2.78 hits per second on day 1 who plays 10 runs of Gridshot per day, skipping no days of practice, would have predicted values of 3.17 hits per second on day 10, 3.29 on day 20, 3.35 on day 30, and 3.39 on day 40. If instead they played 20 runs of Gridshot per day, the same player would have predicted values of 3.22 hits per second on day 10, 3.35 on day 20, 3.42 on day 30, and 3.47 on day 40.

**FIGURE 9:**
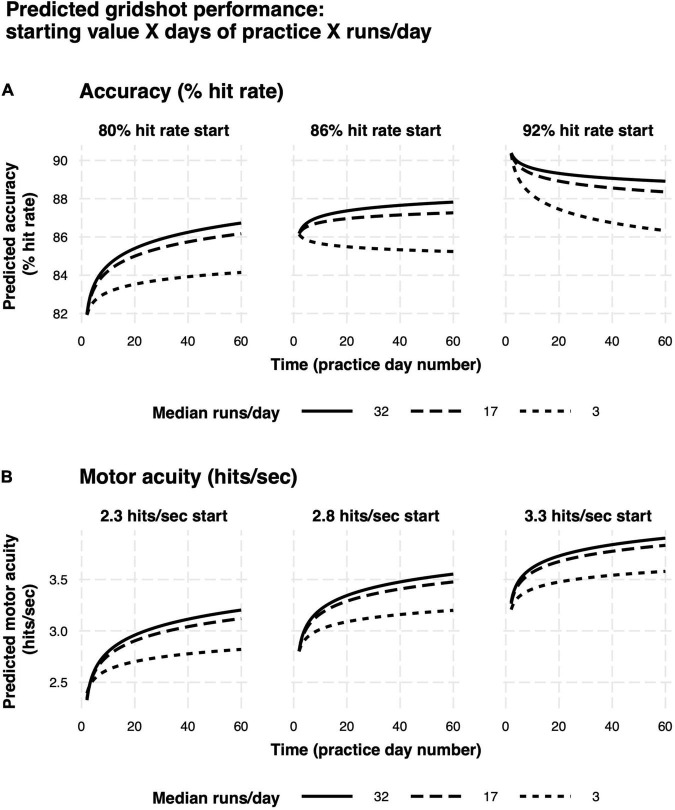
Predicted learning curves. **(A)** Motor performance accuracy (hit rate). **(B)** Motor acuity (hits per second). Different panels delineate model predictions based on differences in baseline motor performance. Curves denote differences in the amount of practice on each day.

Model predictions are summarized in Figures 9A,B. Rate of learning is a function of performance level at the start of learning (hits per second and hit rate start value), median runs of Gridshot per day, and to a lesser extent, number of days between practice sessions. The plots demonstrate the non-linear relationship between additional runs of practice per day and increase in learning per day as well as the non-linear pattern of learning over time, regardless of number of runs per day. In addition, the model of hit rate (Figure 9A) demonstrates that motor performance accuracy is predicted to decrease if it is greater than 88%. The difference between marginal and conditional *R*^2^ for both models (hits per second 0.71/0.93; hit rate 0.26/0.80) estimates the extent to which unmeasured variation among players contributes to variance in each model. A low marginal *R*^2^, in this case, would mean that the model is not very useful in explaining player performance improvement, because a large proportion of variance is due to differences among players rather than measurable predictor variables. For example, the low marginal *R*^2^ (0.26) and higher conditional *R*^2^ (0.80) for the hit rate model indicates that most of the variance in hit rate is explained by differences among players, while most of the variance in hits per second can be explained by the known fixed effects.

**FIGURE 10:**
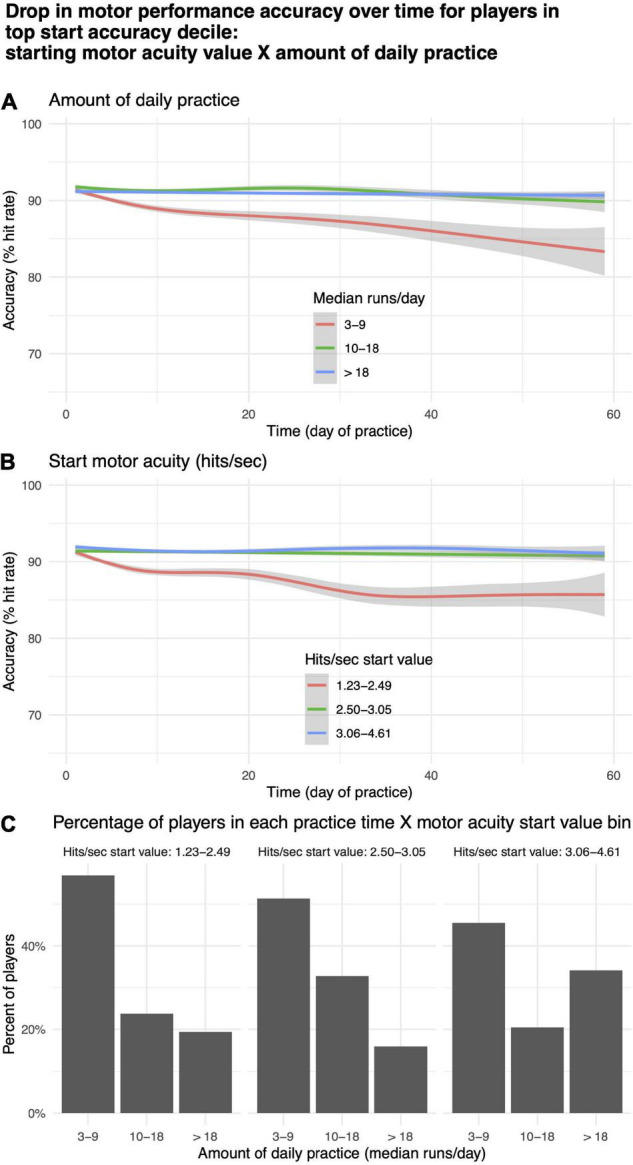
Drop in motor acuity over time for players in top decile. **(A)** By “low,” “medium,” and “high” values for daily practice time. **(B)** By “beginner,” “intermediate,” and “expert” levels for starting value of motor acuity. **(C)** Percentage of motor acuity top decile players within each practice time category, and for each level of starting value of motor acuity.

For a subgroup of players, motor performance accuracy (% hit rate) decreased with practice (Figure 9A, rightmost panel). To explore this phenomenon, we took the top 10th quantile of players based on initial start value for motor performance accuracy (*N* = 714) and split them into tertiles based on median runs per day, corresponding roughly to “low,” “medium,” and “high” values for daily practice time, and start value on day 1 of motor acuity (hits per second), corresponding roughly to beginner, intermediate, expert. Within each of these bins, we plotted the change in motor performance accuracy over time (Figures 10A,B). More than 40% of players in the highest decile of starting accuracy were in the lowest practice time bin (Figure 10C). Those players exhibited a decrease in motor performance accuracy with practice (Figure 10A, red curve). Similarly, those in the lowest tertile for start value of motor acuity (beginners) exhibited a drop in motor performance accuracy over time (Figure 10B, red curve).

## Discussion

While motor learning enables us to successfully interact with our world, it is seldom studied outside the laboratory. As a result, research has focused on how either simple or somewhat contrived motor skills can be quickly acquired in a well-controlled environment (e.g., circle drawing or compensating for mirrored visual feedback (Shmuelof et al., 2012; Telgen et al., 2014; Kasuga et al., 2015; Hadjiosif et al., 2020). Moreover, the population of study is typically homogenous and recruited *via* financial incentives (Henrich et al., 2010). As such, whether these observations from the lab generalize to the acquisition of much more complex real-world motor skills remains an open question. Here, using a first-person shooter game assessment and training platform (Aim Lab), we provide a proof-of-concept of studying motor skill acquisition in a real-world setting. We not only amassed a large sample of motivated gamers who span a wide range of expertise, but also observed how their motor skills progressed longitudinally over 100 days. We found that while their shooting accuracy saturated within a few days, motor acuity continued to show improvements (i.e., shifts in the speed-accuracy tradeoff). Furthermore, these improvements were modulated by the duration of practice, with diminishing returns after roughly 30–60 min of practice.

This proof-of-concept study thus showcases a powerful new approach to study motor learning in the wild, demonstrating in the context of motor learning research, the scientific value of data collected on a large scale in non-controlled conditions (also see: Tsay et al., 2021b). Limitations of this study included factors that were not considered such as Aim Lab tasks the player might have played, other than Gridshot, time of day of gameplay, or number of sessions contributing to a single day of player data. Since Aim Lab does not ask users for personal or demographic information and this study was based on a pre-existing longitudinal data set, factors that could not be taken into account include; time spent playing other video games; player equipment and internet speed differences; health and health behavior-related factors such as prescription or non-prescription drug use, medical conditions or diagnoses, sleep, exercise, or caffeine intake habits, player demographics such as gender, handedness or age, and motivation for using Aim Lab such as training for a competition vs. just for fun.

Although motor learning is comprised of a diversity of learning processes (Kim et al., 2020), a large proportion of studies in the field have focused specifically on motor adaptation in response to visual or somatosensory perturbations, in which the goal of the participant is to return to baseline levels of performance through recalibration of sensorimotor mappings (Krakauer et al., 2019). We hypothesize that the rapid improvement in motor acuity (hits per second) over the first few runs on each successive day (Figure 6B) are attributed to motor adaptation (Shadmehr et al., 2010). Specifically, Aim Lab (like most games of this genre) enables players to set the mouse sensitivity; mouse sensitivity has units of °/cm and determines the amount of rotation in the virtual environment of the game for each centimeter of mouse movement. Since the mouse sensitivity during gameplay is typically incommensurate with mouse usage in other applications (e.g., navigating the desktop to start the game, and navigating the Aim Lab user interface to initiate Gridshot), players may need to recalibrate their movements in response to the new mouse sensitivity setting.

Even though adaptation keeps the motor system well calibrated in the face of changing states and environments (Shadmehr et al., 2010), the goal of movement practice outside the lab, for example, in sports or rehabilitation (Roemmich and Bastian, 2018), is to far exceed baseline levels of performance. Such improvements in motor acuity are accomplished primarily through motor skill acquisition, the gradual improvement in our ability to determine the optimal motor goal, select the appropriate action, and execute a definitive action (Krakauer et al., 2019).

We now consider how our results relate to the three distinct stages of motor learning proposed by Fitts and Posner (1979) (see the section “Introduction”). Motor performance accuracy, as measured by hit rate (i.e., number of targets hit/total shooting attempts), is likely most sensitive to improvements during the cognitive stage. Once the movement requirements are understood – for instance, which keypress to make or how one should sit comfortably in their chair – hit rate may quickly saturate. Indeed, this phenomenon is present in our data, with hit rates reaching a ceiling around 5 days, similar to the findings from previous lab-based motor learning studies (Shmuelof et al., 2012; McDougle and Taylor, 2019). For instance, in visuomotor learning studies where the participant is required to hit the target with their perturbed cursor (e.g., 30° clockwise rotation), performance often reaches an upper bound within a few reaches (Stratton, 1896; Harris, 1963; Kornheiser, 1976; Taylor et al., 2014; Morehead et al., 2015; Hutter and Taylor, 2018; McDougle and Taylor, 2019; Albert et al., 2021; Langsdorf et al., 2021). In other words, participants are quick to figure out how to nullify the perturbation by reaching in the opposite direction. As a result, after these initial trials only marginal improvements in motor accuracy are observed within a single session of practice (Bond and Taylor, 2015; Neville and Cressman, 2018; Huberdeau et al., 2019). The difference in time to saturation between Gridshot and visuomotor rotation (days vs. minutes) likely reflects the increased complexity and task demands of our gaming task.

In contrast to motor performance accuracy, motor acuity, as measured by the number of targets being hit per second, may be more sensitive to improvements during the association stage of motor learning. As evident in our data, hits per second saturates more slowly, and continues to improve for some players over as long as 100 days. Despite performing at a ceiling level in accuracy, players still make considerable improvements in how fast they are moving. Returning to the visuomotor learning literature, a similar analogy can also be made to studies performed in the lab. For example, following introduction of a visuomotor rotation, participants can learn to accurately hit the target with the perturbed cursor, but at the cost of much slower reaction times as compared to baseline reaches (Benson et al., 2011; Fernandez-Ruiz et al., 2011; McDougle and Taylor, 2019; Wilterson and Taylor, 2021). Therefore, even after several days of practice, there remains ample room for improving motor acuity by reducing reaction times (Wilterson and Taylor, 2021). Taken together, unlike cruder measures of motor performance (e.g., accuracy), motor acuity, which takes into account both speed and accuracy, may serve as a more sensitive measure to quantify the progression of motor learning over long periods of practice (Shmuelof et al., 2012, 2014).

This contrast between motor performance accuracy and motor acuity has also been noted by several others (Krakauer et al., 2019). For instance, in a seminal study by Shmuelof et al. (2012, 2014), participants were instructed to draw circles as accurately and quickly as they could within a tight boundary. Improvements in motor skills were quantified by shifts in the speed-accuracy tradeoff function describing the number of circles drawn within a given amount of time. They found that while participants could perform the task relatively accurately within 1 day of practice, improvements in the speed-accuracy tradeoff and coordination (e.g., smoothness of the motor trajectories) required over 5 days of practice. Similar to our study, their measure of motor acuity best captured improvements in motor learning over time (also see: Telgen et al., 2014). Thus, it appears that mastering a motor skill requires prolonged practice over long timescales, whether in the context of learning arbitrary visuomotor mappings (Hardwick et al., 2019), rolling cigars (Crossman, 1959), or, as we show here, gaming.

We were also interested in the critical ingredients that influence the rate of motor skill acquisition. From our exploratory analysis, we found that baseline performance, number of days of practice (day number), median number of runs per day, and the consistency of practice schedule (quantified by number of intervening days between practice sessions) impact improvement in motor acuity and motor performance accuracy (Table 2). Notably, we found that, according to our models, better performance at baseline predicts less improvement with each additional day of practice (Figure 9). Similarly, proficient players benefitted less with additional practice within a given day. A more accurate way to model outcome measures on which there is a ceiling effect is an asymptotic regression as time goes to infinity (Stevens, 1951; Chambers and Noble, 1961). Our implementations of asymptotic regression models indicated that our data collection time period did not extend long enough for player performance to reach an asymptote for either outcome measure. However, our regression models provide operational estimates of the contributions of our predictor variables to outcome measures within the confines of the study time period.

Figure 9 depicts a plot of the predictions of our regression models, rather than data from any one or even a small number of players. So, it is unlikely that any of these effects are driven by statistical outliers such as suggested by the hot hand fallacy (Gilovich et al., 1985). We hypothesize that the predicted decrease in motor performance accuracy for players with high starting accuracy (Figure 9A, rightmost panel) is due to two factors: (1) Some beginner players may place an over-emphasis on accuracy at the expense of speed; and (2) Some expert players may not practice enough to maintain a high level of performance. The decrease in motor performance accuracy over time observed both for players whose daily practice time was lowest (Figure 10A, red curve) and for players in the lowest tertile for start value of motor acuity (beginners or “noobs”) (Figure 10B, red curve) are in agreement with the above two hypothesized factors.

Players who start off with high accuracy but low acuity are “noobs”: they may be moving slowly and deliberately, such that the percentage of their shots fired that hit a target is high. However, the total number of targets they hit within a 60 s run is low. We hypothesize that over time, as they improve, their movements become faster so the percentage of their shots that hit a target may drop, while their overall performance (as measured by score in the game) increases; a drop in motor performance accuracy after a tentative play style on day 1, could be a mark of overall play improvement for noobs.

Players who start off with both high accuracy and high acuity are experts. While moving with high speed, they are simultaneously capable of landing a high percentage of attempted shots on a target. In the case of experts, we hypothesize that a drop in motor performance after day 1 is due to lack of practice and that players who are already experts must devote more time to practicing merely to maintain their current level of performance.

Additionally, we observed diminishing returns on motor learning with increased practice (Rubin-Rabson, 1941; Ryan, 1965; Newell and Rosenbloom, 1981; Figure 8). Specifically, while roughly 1 h of gameplay allows for maximal retention across days, practicing more than this did not improve learning, possibly due to fatigue (Branscheidt et al., 2019). Taken together, our analyses provide several important constraints on motor skill acquisition during video game play, which motivate future experiments to isolate and characterize each factor in detail.

In addition to improvements in motor performance accuracy and motor acuity, we also saw other principles of motor learning in the wild. Specifically, we observed that players retained ∼65% (on average) of their learning from day 1 to day 2. These values are larger than those of previous motor adaptation studies conducted in the lab, with participants retaining ∼40% of their learning over consecutive days of practice (Joiner and Smith, 2008; Telgen et al., 2014). Yet, 65% retention is smaller than retention values following learning a skill *de novo* (i.e., mirror reversal) (Telgen et al., 2014; Wilterson and Taylor, 2021). As such, we speculate that our intermediate retention value reflects the fact that multiple learning systems are engaged during Gridshot, including recalibration of a learned skill as well as acquiring a new skill. We also observed that after an initial drop over the first several days, the percent value of accuracy retention did not decrease drastically over subsequent days. We hypothesize that this is attributable to the small absolute within-day gains in performance accuracy, as players reached a ceiling level of performance accuracy (hit rate) as early as day 3 (Figure 5A).

While there were similarities between the overall approach and findings observed in the present study as compared to many lab-based studies, there were also some fundamental differences that are likely attributable to the differences between tasks. First, tasks used in the lab often involve simple, predictable movements (e.g., center-out reaching, serial reaction time sequence learning tasks, drawing circles). Here, our first-person shooting game, in contrast, requires a complex set of unpredictable movements (e.g., shooting three unknown targets presented sequentially as fast and as accurately as possible). That is, successful gamers not only need to click the correct mouse button and make the appropriate keypresses to shoot, but they also need to immediately plan and execute a set of discrete movements toward unexpected target locations. Arguably, most movements made in daily life are more akin to the longer planning horizons required in first-person shooter games, demanding that multiple actions need to be learned and simultaneously retrieved in short term memory (Gallivan et al., 2018). It is likely these qualitative differences in task demands that contributed to the steady improvements in motor acuity across multiple days of Gridshot practice, rather than quickly hitting a performance ceiling as in most motor learning tasks studied in the lab.

Second, whereas in-lab tasks typically focus on isolating and dissociating one learning mechanism from another (Pellizzer and Georgopoulos, 1993; Hegele and Heuer, 2010; Nikooyan and Ahmed, 2015; Morehead et al., 2017; Kim et al., 2018; Leow et al., 2018; Tsay et al., 2020, 2021a,c; Avraham et al., 2021), our task lies on the opposite end of the spectrum, where a wide range of learning processes are likely involved. For instance, cognitive strategies related to how to play the game (e.g., planning sequences of movements), motor adaptation (recalibrating movements with respect to mouse sensitivity), and skill learning (i.e., increasing the speed of both wrist and finger motions without sacrificing accuracy) all contribute to success in the game. Moreover, gamers are most certainly also learning *via* reward and punishment to determine which movement strategies to retain and which ones to abandon. That is, there is likely an interaction between reinforcement learning and working memory (Collins et al., 2014; Collins, 2017; Collins and Cockburn, 2020). While the analytical tools required to extract how each learning process contributes to motor learning still need to be fully fleshed out, gaming nonetheless provides an ideal way to gather rich data for such multi-dimensional analyses.

Perceptual learning may also contribute to gains in Gridshot performance due to gamers being able to identify and locate relevant targets quickly and accurately. Perceptual learning is optimized through prolonged training, often with significant and long-lasting performance benefits (for reviews, Seitz and Dinse, 2007; Sasaki et al., 2010; Sagi, 2011; Censor et al., 2012; Watanabe and Sasaki, 2015; Dosher and Lu, 2017; Maniglia and Seitz, 2018). The perceptual demands for Gridshot are modest compared to most perceptual tasks (detecting and localizing targets), but the pace at which the task is performed (hitting up to six targets per second) surely stresses the capability of the visual system.

We speculate that ∼85% hit rate optimizes both learning and task engagement. We observed that players exhibit a steady level of performance accuracy (∼85%) throughout learning, even as motor acuity continues to increase due to faster speed (shots per second). One could imagine a different outcome in which performance accuracy increases to nearly 100% without increasing speed. Participants in a previous study likewise voluntarily selected an intermediate difficulty level while learning (Baranes et al., 2014). Errors in learning are beneficial for human learning (Metcalfe, 2017), and 85% accuracy is nearly optimal during the training of machine learning algorithms as well (Wilson et al., 2019). To maintain 85% hit rate while learning, players may adopt a strategy in which they increase their speed slightly, causing accuracy to decrease, and then practice at that new speed until their performance accuracy recovers before increasing their speed again.

The future of studying motor learning through video games is promising (Anguera et al., 2013; Stafford and Dewar, 2014; Chen et al., 2018; Stafford and Vaci, 2021; Tsay et al., 2021b). Gaming provides a unique medium and potentially large data sets to investigate how motor skills are developed through intrinsic motivation, rather than financial incentive (Aung et al., 2018). For instance, previous studies examining motor learning during neurorehabilitation have suggested that intrinsic motivation is a critical ingredient for motor retention (Kleim and Jones, 2008; Winstein et al., 2014; Winstein and Varghese, 2018; Tsay and Winstein, 2020). The statistically significant within-player performance difference between Gridshot runs played in practice (scores visible only to the player) vs. compete (scores visible to other players) modes in this study demonstrates the effect that motivation can have on player performance in a motor learning task. In a similar vein, gaming naturally lends itself to longitudinal studies, rather than being limited to cross-sectional studies (Crossman, 1959), affording greater within-subject controls and insights into the individual differences of motor expertise (Anderson et al., 2021). Gaming also provides a convenient way to amass a wealth of moment-to-moment kinematic data. Recent studies have shown how much kinematics can reveal, ranging from variables related to decision making (Song and Nakayama, 2008, 2009; Freeman and Ambady, 2010) to those related to one’s reward expectations (Summerside et al., 2018; Sedaghat-Nejad et al., 2019). Future iterations of Aim Lab task-based studies will focus on collecting and analyzing these fine-grained kinematic data, which will provide deeper insights into how complex motor skills can be acquired and refined in an ecological setting, outside the traditional laboratory.

## Data Availability Statement

The datasets presented in this article are not readily available because the raw data for this manuscript were acquired for commercial purposes. The data are not scheduled to be made publicly available because they are proprietary. Requests to access the datasets should be directed to research@statespacelabs.com.

## Ethics Statement

Data were initially acquired for commercial purposes and are stored without personal identifiers; thus, informed consent is not required for this study (Advarra Institutional Review Board, Columbia, MD, United States).

## Author Contributions

DH assisted with study design and manuscript preparation. WM designed and implemented the study apparatus and tasks. JL carried out data analyses and assisted with study design and manuscript preparation. HK and JT assisted with manuscript preparation. All authors read and approved the final manuscript.

## Conflict of Interest

JL and WM are employed by Statespace Labs. DH is a paid consultant for Statespace Labs. This study received funding from Statespace Labs. The funder had the following involvement with the study: The funder developed and maintains the commercial software and database for the free video game, Aim Lab, which served as the data collection instrument for this study and all paid publication fees. All authors declare no other competing interests.

## Publisher’s Note

All claims expressed in this article are solely those of the authors and do not necessarily represent those of their affiliated organizations, or those of the publisher, the editors and the reviewers. Any product that may be evaluated in this article, or claim that may be made by its manufacturer, is not guaranteed or endorsed by the publisher.

## Footnotes

1
https://www.youtube.com/watch?v=dF3AYbE810Y

## Supplementary Material

Supplementary File 1 Sample R code for regression analysis of motor learning over time.

Supplementary File 2 Simulated train data for use with sample R code, train_set_sim.csv.

Supplementary File 3 Simulated test data for use with sample R code, test_set_sim.csv.
