# Supplementary material for: Long-Term Motor Learning in the “Wild” With High Volume Video Game Data
Source: Front Hum Neurosci. 2021 Dec 20;15:777779. doi: 10.3389/fnhum.2021.777779 (PMC8720934; doi:10.3389/fnhum.2021.777779)

Simulated Gridshot Motor Learning Regression Modeling

Jenny Listman / Statespace Labs

11/23/2021

Sample code for regression analysis of motor learning over time in "Long-term Motor Learning in the Wild with High Volume Video Game Data" by Jennifer B. Listman, Jonathan S. Tsay, Hyosub E. Kim, Wayne E. Mackey, David J. Heeger

```
# load R packages

library(readr)
library(tidyverse)
library(lmerTest)
library(insight)
library(performance)
library(interactions)
```

There are vast resources online for learning how to develop and test mixed effects, repeated measures models in R. This is not a tutorial and assumes prior knowledge of R. Unfortunately, there is no single correct work flow for this process because it is heavily dependent on the specific outcome variable and predictor variables involved and requires numerous judgment calls on the part of those conducting the analysis. Choosing predictor variables, including removing variables that are highly correlated, is a separate process. Variable scaling, transforming, and centering is an entire topic, on its own. In addition, the process you choose could depend on the goal of model development - are you building a predictive algorithm or conducting an exploratory analysis to characterize how or if various factors contribute to your outcome measure? Here, we conducted an exploratory analysis. Either way, a lot of time should be devoted to exploratory data analysis and data visualization prior to model development and testing.

After exploratory data analysis and visualization, we chose to log transform time (in the original data set, day_number) and amount of daily practice (in the original data set, median_runs).

The data set used here is a dummy repeated measures data set, not produced from Aim Lab, with different start values, number of data points, and amount of practice per time unit per subject; variable names have been changed to protect the innocent and units have been scaled arbitrarily. Data has been divided (80/20) into train (N = 1840) and test (N = 459) sets, balanced for total number of practice sessions (n_time_subject) per subject.

```
# read in data
test_set_sim <- read_csv("test_set_sim.csv")
train_set_sim <- read_csv("train_set_sim.csv")

# change subject_id from numeric to factor variable

test_set_sim <- test_set_sim %>%
  mutate(subject_id = as.factor(subject_id))
```

```r
train_set_sim <- train_set_sim %>%
  mutate(subject_id = as.factor(subject_id))

# view summary of data
summary(train_set_sim)
```

```
##    subject_id         time        time_skipped     n_time_subject
##  22     :   59   Min.   :0.100   Min.   : 0.1000   Min.   : 5.00
##  98     :   59   1st Qu.:0.400   1st Qu.: 0.1000   1st Qu.:11.00
##  103    :   59   Median :0.900   Median : 0.1000   Median :21.00
##  104    :   59   Mean   :1.371   Mean   : 0.4909   Mean   :26.35
##  158    :   59   3rd Qu.:1.900   3rd Qu.: 0.3000   3rd Qu.:39.00
##  168    :   59   Max.   :5.900   Max.   :19.5000   Max.   :59.00
##  (Other):28346
##  median_practice_amount start_performance daily_performance
##  Min.   :-0.1662        Min.   :0.09133   Min.   :0.03537
##  1st Qu.: 0.6757        1st Qu.:1.22256   1st Qu.:1.66115
##  Median : 1.1681        Median :1.51650   Median :2.00881
##  Mean   : 1.5531        Mean   :1.54887   Mean   :2.01162
##  3rd Qu.: 1.9661        3rd Qu.:1.86701   3rd Qu.:2.34679
##  Max.   :14.5056        Max.   :3.62344   Max.   :3.98492
##
```

```r
summary(test_set_sim)
```

```
##    subject_id        time        time_skipped     n_time_subject
##  55     :  59   Min.   :0.100   Min.   : 0.1000   Min.   : 5.00
##  119    :  59   1st Qu.:0.400   1st Qu.: 0.1000   1st Qu.:12.00
##  448    :  59   Median :1.000   Median : 0.1000   Median :22.00
##  474    :  59   Mean   :1.405   Mean   : 0.4683   Mean   :27.09
##  903    :  59   3rd Qu.:2.000   3rd Qu.: 0.4000   3rd Qu.:42.00
##  1303   :  59   Max.   :5.900   Max.   :16.7000   Max.   :59.00
##  (Other):6962
##  median_practice_amount start_performance daily_performance
##  Min.   :-0.1827        Min.   :0.4058    Min.   :0.3058
##  1st Qu.: 0.6517        1st Qu.:1.2676    1st Qu.:1.7096
##  Median : 1.1151        Median :1.6158    Median :2.0546
##  Mean   : 1.5477        Mean   :1.6135    Mean   :2.0710
##  3rd Qu.: 2.0023        3rd Qu.:1.9106    3rd Qu.:2.4244
##  Max.   :12.3954        Max.   :3.4543    Max.   :3.8197
##
```

The maximal model, including all possible interactions, is written out in `lmer` format

In this case, our model:

- assumes independent slope (learning rate) and intercept (starting value of performance measure) per subject.
- includes interactions among all possible predictor (fixed effect) variables

‘

```r
# write maximal model

big_model <- daily_performance ~ start_performance*log(time)*log(median_practice_amount)*time_skipped +
  (1 + log(time) | subject_id)
```

1. Fit the linear mixed effects regression model with `lmerTest::lmer`.
2. Use stepwise elimination with `lmerTest::step` to remove variables or interactions from the maximal model that do not contribute significantly to the model.
3. Use `summary` to view model coefficients (note that the final model excludes some interaction terms present in the maximal model after `lmerTest::step`)

The `insight::find_algorithm` , `insight::model_info` , and `insight::get_variance` functions can be helpful for accessing `lmer` settings you might want to report in a manuscript.

```r
# fit maximal model

fit <- lmerTest::lmer(big_model, data=train_set_sim)

# backward elimination using terms with default alpha-levels

step_fit <- lmerTest::step(fit)

# extract final model

final_fit <- lmerTest::get_model(step_fit)

# view summary of model

summary(final_fit)
```

```
## Linear mixed model fit by REML. t-tests use Satterthwaite's method [
## lmerModLmerTest]
## Formula:
## daily_performance ~ start_performance + log(time) + log(median_practice_amount) +
##     time_skipped + (1 + log(time) | subject_id) + start_performance:log(time) +
##     log(time):log(median_practice_amount) + start_performance:time_skipped +
##     log(time):time_skipped + start_performance:log(time):time_skipped
##    Data: train_set_sim
##
## REML criterion at convergence: -20737.4
##
## Scaled residuals:
##     Min      1Q  Median      3Q     Max
## -7.8847 -0.5385  0.0410  0.5972  4.9194
##
## Random effects:
##  Groups     Name        Variance Std.Dev. Corr
##  subject_id (Intercept) 0.05925  0.2434
##             log(time)   0.00980  0.0990   0.67
##  Residual               0.02114  0.1454
## Number of obs: 28391, groups:  subject_id, 1815
```

```
##
## Fixed effects:
##                                         Estimate Std. Error         df
## (Intercept)                            7.941e-01  2.009e-02  1.750e+03
## start_performance                      7.750e-01  1.257e-02  1.740e+03
## log(time)                              2.785e-01  9.377e-03  1.786e+03
## log(median_practice_amount)            5.331e-02  6.153e-03  1.763e+03
## time_skipped                          -5.959e-03  3.196e-03  2.593e+04
## start_performance:log(time)           -7.099e-02  5.843e-03  1.749e+03
## log(time):log(median_practice_amount)  1.986e-02  2.870e-03  1.808e+03
## start_performance:time_skipped        -3.573e-03  1.917e-03  2.597e+04
## log(time):time_skipped                -1.517e-02  2.898e-03  2.613e+04
## start_performance:log(time):time_skipped  4.948e-03  1.856e-03  2.626e+04
##                                       t value Pr(>|t|)
## (Intercept)                            39.525  < 2e-16 ***
## start_performance                      61.635  < 2e-16 ***
## log(time)                              29.696  < 2e-16 ***
## log(median_practice_amount)             8.664  < 2e-16 ***
## time_skipped                           -1.865  0.06226 .
## start_performance:log(time)           -12.150  < 2e-16 ***
## log(time):log(median_practice_amount)   6.918 6.32e-12 ***
## start_performance:time_skipped         -1.864  0.06229 .
## log(time):time_skipped                 -5.236 1.65e-07 ***
## start_performance:log(time):time_skipped   2.665  0.00769 **
## ---
## Signif. codes:  0 '***' 0.001 '**' 0.01 '*' 0.05 '.' 0.1 ' ' 1
##
## Correlation of Fixed Effects:
##             (Intr) strt_p lg(tm) lg(__) tm_skp st_:() l():(_ str_:_ lg():_
## strt_prfrmn -0.954
## log(time)    0.651 -0.619
## lg(mdn_pr_)  0.058 -0.072  0.036
## time_skippd -0.110  0.101 -0.118  0.006
## strt_prf:() -0.622  0.651 -0.953 -0.047  0.108
## lg(tm):(__)  0.035 -0.046  0.055  0.662  0.006 -0.073
## strt_prfr:_  0.106 -0.107  0.112 -0.003 -0.953 -0.114 -0.003
## lg(tm):tm_s -0.044  0.036 -0.189 -0.001  0.481  0.176 -0.008 -0.450
## strt_p:():_  0.035 -0.032  0.172  0.002 -0.415 -0.177  0.012  0.433 -0.950
```

```
# insight::get_variance(final_fitl) # useful for reporting
# insight::find_algorithm(final_fit) # useful for reporting
# insight::model_info(final_fit) # useful for reporting
```

How well does the model generalize? Compare RMSE obtained from train vs test sets. If RMSE is much worse using the test set, then the model could be over-fitted. In this case, the model doesn't appear to be over-fitted.

Mixed-effects models employ both marginal and conditional R^2 metrics, where marginal R^2 refers to the amount of variance explained only by fixed effects (here, fixed effects refers to time, start_performance, time_skipped, and median_practice_amount) and conditional R^2 refers to the amount of variance explained after adding in random effects (here, random effects refers to varying intercept and slope among individuals).

```
fit_train <- final_fit

# compare model performance with test vs train sets

fit_test <- lmer(fit_train, test_set_sim)

performance::compare_performance(fit_train, fit_test, metrics = "common")
```

```
## # Comparison of Model Performance Indices
##
## Name      |           Model |        AIC |        BIC | R2 (cond.) | R2 (marg.) |   ICC |  RMSE
## -------------------------------------------------------------------------------------------------
## fit_train | lmerModLmerTest | -20709.357 | -20593.803 |      0.924 |      0.690 | 0.756 | 0.138
## fit_test  | lmerModLmerTest |  -5227.801 |  -5131.347 |      0.921 |      0.682 | 0.752 | 0.138
```

```
# predict scores in the test set

predicted_daily_performance <- predict(fit_train,
  newdata = test_set_sim,
  random.only=FALSE,
  allow.new.levels = TRUE)

# add predictions to test set

test_set_sim[["predicted_daily_performance"]] <- predicted_daily_performance

# plot actual vs predicted

ggplot(test_set_sim, aes(daily_performance, predicted_daily_performance)) +
  geom_point() +
  geom_smooth() +
  coord_equal() +
  ggtitle("test set: predicted vs actual")
```

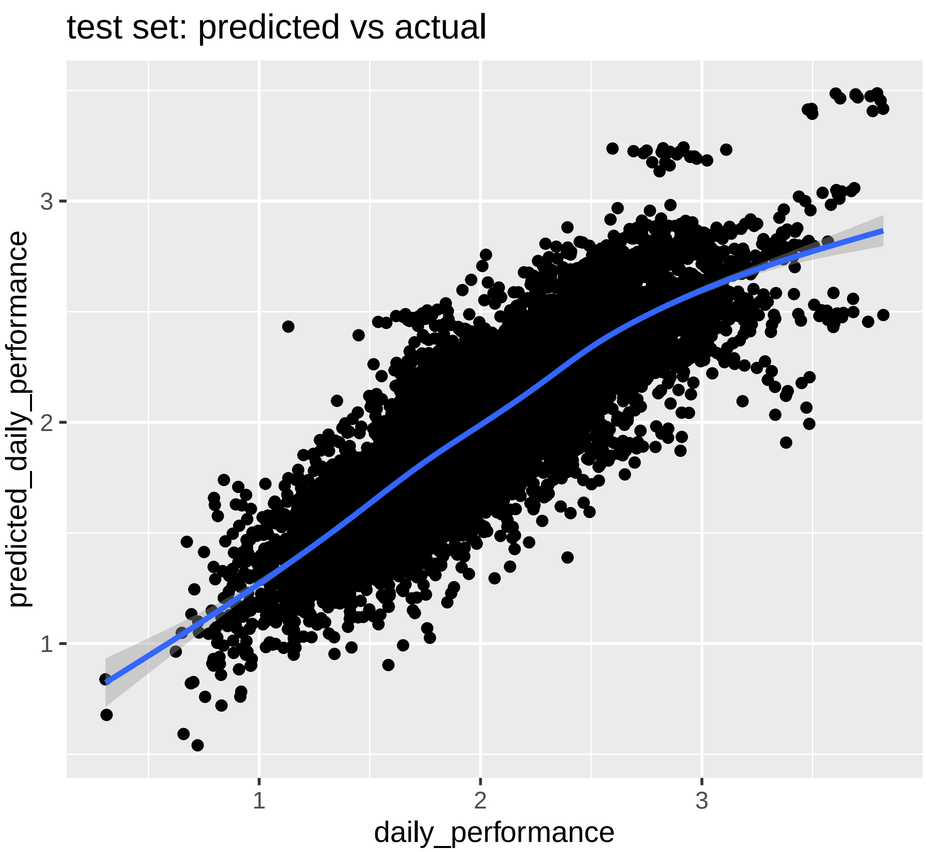

Trying to make sense of a table of coefficients can be difficult. Sometimes it helps to visualize model effects.

Use `interactions::interact_plot` to visualize the model effects.

```
# does the model make any sense?
# plot some interactions instead of just reading the table of coefficients

interactions::interact_plot(final_fit,
                            pred = time,
                            modx = median_practice_amount,
                            mod2 = start_performance
                                                 )
```

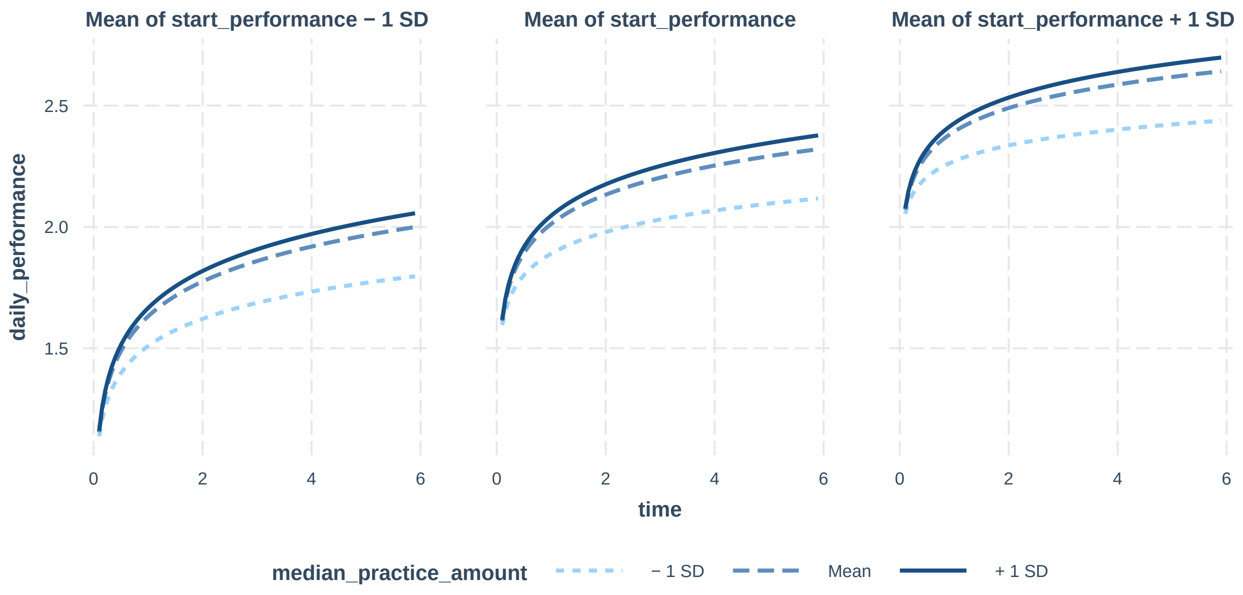


```r
# plot more

interactions::interact_plot(final_fit,
                            pred = time,
                            modx = time_skipped
                            )
```

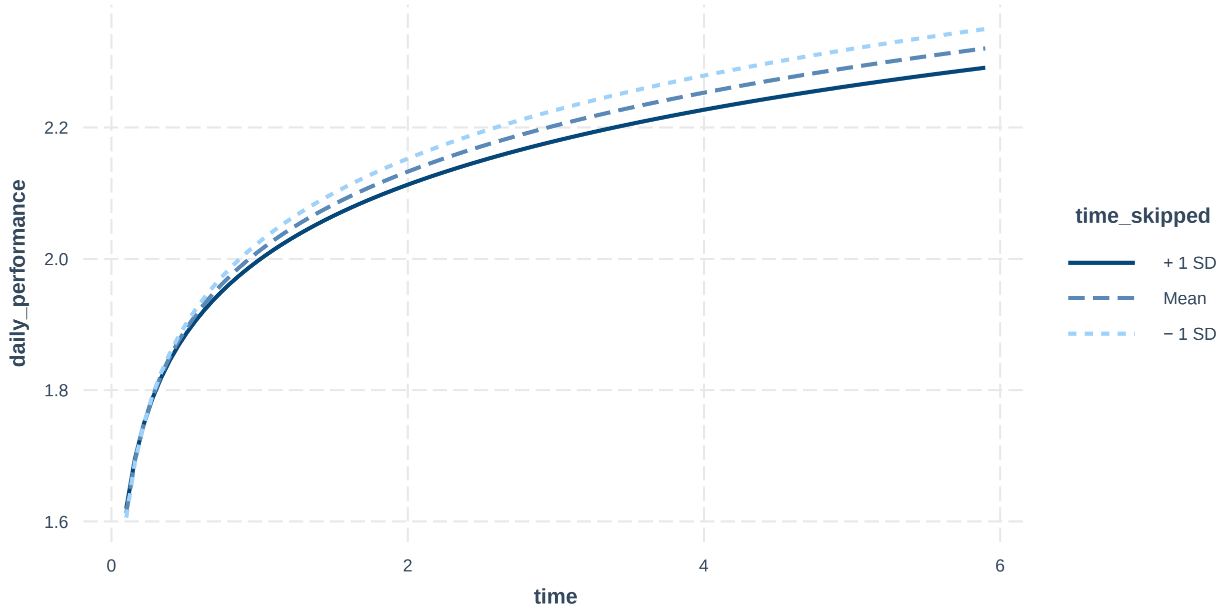

Supplement: Supplementary File 1 — Sample R code for regression analysis of motor learning over time. [file Data_Sheet_1.PDF]
